# Research on Constructing a Healing Environment for the Street Spaces of a High-Density City: Using Street Spaces in Macao’s Old City Area

**DOI:** 10.3390/ijerph17134767

**Published:** 2020-07-02

**Authors:** Lingchao Meng, Chun Zhu, Kuo-Hsun Wen

**Affiliations:** 1 Faculty of Humanities and Arts, Macau University of Science and Technology, Avenida Wai Long, Taipa 999078, Macao; lcmeng87@gmail.com; 2 School of Arts, Macau Polytechnic Institute, Taipa 999078, Macao

**Keywords:** high-density city, street spaces, healing environment

## Abstract

It is commonly recognized that street spaces in high-density cities are able to cause negative impacts in terms of residents’ physical and mental health. This research intends to investigate and analyze how residents use street spaces in a high-density city in order to construct a healing environment for these street spaces. The research was conducted in Macao’s old town by using spatial syntax methods to define the research areas, and implemented on-site observations that evaluated the age of the residents in the space and the conditions of their usage of the space. The study collected data through expert grading and employed the Analytic Hierarchy Process to calculate the weight of each indicator in order to attain accurate and objective research outcomes. The evaluation results indicate that the current Macao street spaces are poor healing environments. By analyzing the effective factors for constructing a healing environment in these street spaces, so that residents can get more space for healing when they use it, the paper aims to provide a model example for those who are involved with city governance, planning and design.

## 1. Introduction

Street spaces and the residents’ behavior and health can be regarded as a sort of coupling relationship. Encountering an increasing population and the need for multiple uses of spaces in cities, streets may be seen as not only paths for transportation but also useful to fulfill functions of public places for daily life. On the streets, residents can develop various activities, such as socialization and recreation, where health can be attained. The design and management of street spaces require the collaboration and implementation of a multidisciplinary team. Therefore, global organizations, such as the World Health Organization, began to pay attention to the relationship between urban social culture and ecological environments that are also key determining factors to prevent epidemic and non-epidemic diseases. However, for researchers, their studies need to consider a wider range of influential factors [1].

Macao is a typical high-density city, and before 1999, Macao was administered by Portugal. At the beginning of the 20th century, in some blocks, the Macao Portuguese government adopted the European rectangular system of city planning to Macao’s old city area where streets are usually very narrow in width (less than 10 m) and the layout of the street networks appears as complex, non-linear or curved patterns, which is suitable for pedestrians or animal transport. Most of the buildings’ height is less than 20 m. The ratio between some buildings and roads is less than 1:1.5 m, revealing a tidy and beautiful scene that makes the streets adapted to the Macao climate, where the airflow can go through all the building blocks and meet the needs of sufficient sunlight in the building to the greatest extent, thereby reducing the chance of germs breeding [2]. The population distribution of Macao is dominated by the Peninsula, and its area is around 9.3 km^2^; that is, 80.6% of the population is concentrated on 30.7% of the total land area, where its population is unevenly distributed. According to statistics for 2015, the population density reached 21,100 people per square kilometer. Consequently, the daily lives of most Macao people are implemented in crowded street spaces where people have to get more space for activities and at the same time assure their own safety. It is very precious for people to have their social activities and entertainment, demonstrating a course that meet people’s authentic needs in the outdoor environment, as well as acquiring health. However, with an increasing population, aging people, tourists and the numbers of motorcycles and automobiles, those poor street spaces have been eroded continuously.

### 1.1. Street Space and Residents’ Behavior

Usually, it is recognized that the various types of activity content in spaces are identified by user behavior, such as communication, walking, sitting and standing, etc. [3]. The combination of physical elements between spaces and their comforts is the assurance of inducing various behavioral activities and attaining resident health [4,5]. In previous studies, researchers tried to find relationships between a street view (Google Street View, Baidu Street View) and residents’ health [6,7,8]. It includes the occupancy of street vendors [9,10]; pedestrian environments or street “microscale” characteristics, including seating, lighting, street furniture, pavement characteristics, the height of the adjacent buildings, street width and intersection design [11,12,13,14]; and environmental comfortability [15] and quality. Improving the street space environment, such as the space layout and connectivity, so as to attract more pedestrians, has a positive role for the pacers, thereby improving traveler satisfaction and so on [16,17,18].

Social activities in the street spaces, i.e., interactions between residents and between residents and the spaces [19], even if only adding small changes a resident’s lifestyle and behavior, can have an impact on individual health and social well-being. For example, in Tokyo, there are illustrations drawn on street surface in a three-dimensional way, which are used for traffic control or the obstacle to affect the driving behavior where the safer street environment can be achieved for pedestrians [20]. The “HK Regeneration” plan attempts to provide comfortable facilities for residents in empty street spaces that do not have any function and restore abandoned street spaces in the city for life revitalization [21,22]. In Amsterdam, a proposed plan in is employed to reduce parking spaces year by year and utilize free parking spaces for trees and sidewalks [23]. Similarly, the urban cultural background will affect the age and group size of the users in the street space, and the content of the space used and the location of the space occupied by the three types of social relations (intimate group, intimate group and social group) will be reflected differently under the influence of different urban cultures [24].

As indicated in previous studies, it has been confirmed that the space environment plays a vital role in supporting residents to implement various behavioral activities and ensure more health perceptions during the process of use. However, for those streets in high-density cities, such as Macao, functionality in the space itself is rather overlapped and complicated. It would not be sufficient to carry out research only from a single perspective. In fact, it is necessary to proceed with a series of systematic research.

### 1.2. Healing Environment

Being one of the representative theories of environmental psychology research on the interactions between the human and physical environment, the healing environment theory provides useful references for urban design, environmental design and public health from rationality and experience. A Kaplan couple identified that in an environment with “distant, charming, extensibility and compatibility” characteristics, any individual could recover from their declining concentration ability effectively [25]. “Stress Reduction Theory” by Ulich and “Attention Restoration Theory (ART)” by Stephen and Rachel Kaplan together provide strong support for the viewpoint of environment that has healing effects [26]. Gesler reckons that the built environment, social conditions and human concepts have created an atmosphere conducive to rehabilitation. After reviewing research on healing environments [27], Van den Berg pointed out that nature, light, fresh air and quietness are four classic environmental factors to be extended to more space types [28]. In addition, Malkin addressed whether some characteristic factors in the physical environment can be regarded as the environmental factors with healing effects. However, other non-physical factors may still be of equal importance for creating a healing environment [29]. OHE is such a conceptual framework, created by the SAMUEL Institute, which encompasses relationships, creating healthy behaviors, as well as the physical environment around you [30]. Furthermore, standing on the viewpoint without considering designers or developers, Huisman investigated and conducted design research based on end-user needs [31]. Dubose reviewed the existing evidence for spatial facilitation of healing based on Evidence-Based Design (EBD). Environmental variables that directly affect or promote one or more dimensions were enriched into six groups of variables [32].

As a result, the healing environment theory and its scope of application are extending continuously. Fei Xue reinterprets the transformation of the healing space in its contemporary context by focusing on the sensory level of healing perception, and formulates a conceptual framework that can enhance the performance of the healing space, emphasizing the supportive environment and sensory perception for healing and recovery importance. Leiqing Xu has shown that both the green vision rate and the street interface have a significant impact on the street healing [33,34]. Therefore, if street spaces were provided with healing functionality by a reasonable layout, it could fulfill residents’ needs for having specific functionality and at the same time attain the psychological effects of healing. Based on such a perspective, street spaces would become an effective carrier that matches the goal of street spaces with multiple spatial functionalities for high-density cities like Macao.

### 1.3. Urban Renewal Policy of Macao

Policymaking is in a way to secure the development and keeping of new spatial science updates. In the development process of Macao, limited land area has always been one of the key factors restricting its effective renewal. Under the premise of this argument’s point and evidence, in 2006, the Macao Sustainable Development Strategy Research Center proposed relevant strategies, goals and public policies to be correctly formulated and coordinated for promoting things that are closely related to making resident’s change over a short time [35]. However, it can be difficult to make accurate judgements about the priority of handling these things that are closely related. For example, in 2007, government administrators resolved the shortage of motorcycle parking spaces for residents. By providing 7000 permanent and 4000 temporary parking spaces for cars and motorcycles within three months, it actually puts a greater burden on the original shortage of land resources and urban space, especially on the peninsula, which limits the flexibility of space development [2]. As of 2014, the Macao island road has a total length of 424.2 km; so locally Macao could have a total of 240,107 cars, if the calculation is based on the average distance between every car conductor being 4 m. Macao motor vehicles have a total length of 960.4 km; it is 2.3 times the length of the road in Macao, and the street space is overloaded [36]. In this serious situation, how to use street space effectively is a challenge for residents, designers and managers. The Macao SAR established the Urban Renewal Commission in 2016 with the aim to improve the living environment for residents of the old district, which is also the basic content of the future Macao development [37]. The Macao SAR government has urged to pay attention on matters that are closely related to the residents and make efforts for improving the living environment in the old city area.

In summary, this study emphasizes the need for a comprehensive framework to consider how to implement and more accurately and effectively construct street space renewal and healing functions. Ideally, this will guide practitioners and decision-makers to consider the impact of residents’ spatial behavior on their health as they undertake design updates. In the following chapters, the study applies DepthMap X software to determine the observation area through space syntax, conducting space observations on selected spots, using field data, combining activities and space occupations, and to define various factors connected to the residents’ usage. Based on the content of the “healing environment”, the building healing environment for Macao’s street spaces is first analyzed, and then the mechanism of refining future research and renewal design practices are both discussed.

## 2. Research Materials and Methods

This research uses mixed application of quantitative and qualitative methods through space syntax to find street spaces with high integration and connectivity among the research areas. Typically, these spaces are more easily accessible to residents, and provides a more accurate understanding of the residents’ use of the street spaces. Attention should be based on the space syntax of the spatial layout analysis that was used for the prediction index of space connectivity and spatial behavior. However, due to a series of external factors, such as social culture, economy and population characteristics, the results calculated by simulation software cannot fully reflect the actual situation. In order to systematically analyze and evaluate the concentration and process of resident activities and the priority of building healing environment contents in street space, attention should be paid to the relationship between multiple variables. At this time, field observation is usually used as a field investigation method to enrich and strengthen field research and establish relationships between different variables.

The specific implementation steps are as follows:

1. Implementing space syntax analysis to the street spaces of all research areas according to connectivity and integration;
2. Conducting on-site observations and classification records of residents’ space usage by using age and activity types for summarizing and comparing;
3. To classify recorded images containing activities in spaces for better categorization;
4. Applying environmental factors of healing functionality [29] and Optimal Healing Environments (OHE) [30] to construct the healing environment of street spaces in Macao; data used in the paper were attained from on-site investigations, questionnaires, expert scoring as well as the Analytic Hierarchy Process (AHP) and Fuzzy Comprehensive Evaluation (FCE) for detailed analysis.

### 2.1. Area Selection of the Research Unit

The research unit located in Macao’s old town has strong representativity. The street space is very narrow, and the population type is mainly local residents, foreign workers and tourists.

**Figure 1:**
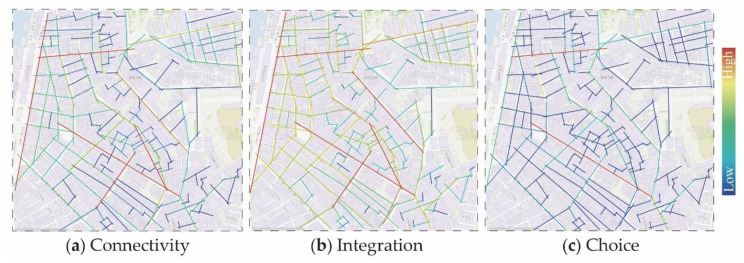
Analytic picture of the space syntax. (**a**) Connectivity; (**b**) Integration; (**c**) Choice.

**Figure 2:**
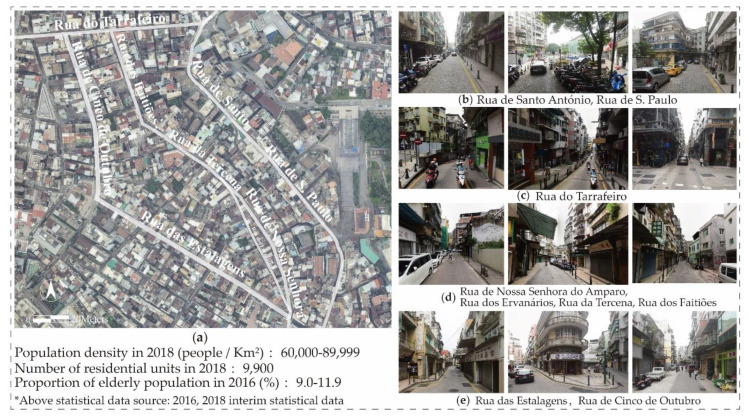
Picture of the research area and street scene. (**a**) Details; (**b**–**e**) Ruas.

For the analysis of the connectivity of the space syntax to the research area, Rua do Tarrafeiro, Rua das Estalagens, Rua dos Ervanários, Rua de Camile Pessanha and Rua das Lorchas are streets with the most connection points (see Figure 1), implying that these street spaces are easier to enter and leave. The connectivity value of Ruínas de São Paulo is 10; Rua de Cinco de Outubro’s connection value is 8; Rua da Tercena and Rua de Nossa Senhora do Amparo’s connection values are 7; and Rua de Santo António and Rua dos Faitiões are both at 6. Through overall data observation analysis, the connectivity values of 2 and 3 occupy most of the street spaces in the research area and the second is the street space with connectivity values of 5 and 6. This is consistent with the current situation of the street space in the area. Most of the street spaces are short and narrow and mainly are used for passages that are difficult to make space for gatherings. By combining the results with the scene and further analyzing figures of “Integration” and “Choice”, “Integration” can be interpreted as the degree of aggregation or dispersion of a space with other spaces, and “Choice” is defined as measures of movement flowing through spaces, which is a powerful measurement on the forecasting potentials of pedestrian and vehicular movement. With removing the street space used for the passage of motor vehicles, we selected Rua de Santo António, Rua de S. Paulo, Rua do Tarrafeiro, Rua dos Faitiões, Rua do Minho, Rua da Tercena, Rua de Nossa Senhora do Amparo, Rua dos Ervanários, Rua das Estalagens and Rua de Cinco de Outubro as the practical spaces for investigation and research (Figure 2).

### 2.2. Observation Phases

Observation methods has been used as an effective way for researchers to understand the interactions between a physical space and humans, as well as between humans. In Whyte’s “The social life of small urban spaces”, the author employed observation methods to analyze the spatial elements (sun, tree, bench, etc.) connecting with people. He emphasized the effectivity of small spaces, such as redundant and recessed spaces [38]. When observing a space, it can be realized by how it works practically but not how to use it. Gehl employed eight methods, such as an on-site counting method, map marking method, image recording method, etc., as systematic research tools for gaining the interactive observations between spaces and behavior. Furthermore, it emphasizes tool selection by referencing factors such as research purpose, time and climate. Through the use of patterns of static photography recording, it is effective for both static and dynamic activities, and it discovers more potential interactions that occur naturally [5].

Based on previous study results and methods implemented by researchers, this paper applies the map marking method and image recording methods. The combined method owns a higher cost and time effect and attains useful information that is relevant to designing spaces eventually [3,39]. The observational phases of the research are aimed towards different time and weather conditions in an entire day. To avoid a discrepancy in the observation record of the resident’s activity, the paper defines activity as the activity of any particular individual, such as residents participating in two activities at the same time or residents stopping one activity and starting another. All those related activities were recorded within an observed time length. To achieve the best efficiency, we used different symbols to mark the location on the map, and recorded the activities of the residents and the content of the space environment directly on the behavior map, and took photos to record them.

The five steps in the observation process are the following:

1. The mapping of the observation area;
2. Defining the exact definitions of the observations, descriptions or graphics of the public behaviors;
3. A repeated time arrangement for observations and records during the set period;
4. Systematic recording of the process of the observed behavior;
5. Be familiar with the use of a coding and counting system that can minimize the required workload for recordings and observations.

### 2.3. Acquisition of Behavioral Data

The time period for collecting research data and observations was March to April, 2019. There were three time-intervals of observations per day (7 a.m. to 10 a.m., 11 a.m. to 3 p.m., 5 p.m. to 7 p.m.). For on-site observations, we used connectivity and integration based on the space syntax methodology to observe the selected streets. The observation time for each street was 30 to 50 min continuously, and there were 30 copies of the collected data samples. The on-site observations were implemented under good weather conditions that attracted residents to comfortably use the street spaces.

Different symbols were set for on-site observations to indicate the location and activities engaged in crowd or individual gatherings. It also tried to match age and activity types with those set symbols to ensure the validity of the data completeness and accuracy. Age groups were classified as children (under 15 years old), youth (around 15 to 44 years old), middle-aged (around 45 to 55 years old) and the aged (over 65 years old); however, it does not include by-passes or stays with short time (less than 5 min), such as walking, because the key point of the study is mainly focusing on long-time gatherings of residents and those environments supporting the implementation of the gathering activities.

This study assumes that those residents gathered in the spaces are acquainted with each other or have common needs for space. Results of the observations are classified into 4 types:

1. Chatting (seated), a scene intended as communicating with each other, comfortable, pleasant, etc.;
2. Chatting (standing, leaning on), a scene described as chatting with friends or family;
3. Waiting (standing, leaning on), a scene expressing anxiety, relaxation, etc., in the same place with known individuals or strangers;
4. Travelers (seating, standing, leaning on) who are curious about the space, a scene expressed as pleasure, relaxation, anxiousness, etc.

In previous studies, most of them applied the same methods to implement on-site observations in groups. However, observational results of this study were implemented by two research members. For assuring the completeness of study, the observing locations for the on-site observations were simpler and more concentrated. Eventually, the study conducted age evaluation in a predetermined range based on common recognition, and implemented identification and separate records of gender, activity and the content of the environment. According to the observed data, the study obtained the age range and gathering time of people engaging with activities in the street space (using day and week as time unit, day unit was classified as morning, noon and night; and week unit was classified as working day, weekend and holidays). The descriptions above were used as the basis of the data analysis and model construction.

### 2.4. Analysis of Behavior Data

The observational data collected during this study included 106 observation times and 957 residents. There were 301 individuals in the collected data and 78 observation times applied to the crowd. The records also remarked on other relevant information during the observation phase. The age and number of gathering people is a quantitative value. Activities and the content of the spatial environments were recorded as descriptive terms, such as sitting, standing, leaning, talking and shopping. Through marking on maps with different symbols occupying the spaces, the data recordings were implemented on printed observation maps and then keyed into MS Excel spreadsheets for further classification analysis.

It is complicated to record the content of residents’ activities among their gatherings, such as sitting, talking, eating, standing, chatting and shopping, which shows a certain similarity to the content of the gatherings in these spaces. Afterwards, 12 various activities were confirmed, including sitting, chatting, playing, shopping, selling, eating, calling on mobile phones, playing telephone, looking around, taking photos, music listening, reading and exercising. The next step is that the spatial contents of those activities were recorded separately and categorized as collected data for simplification, for example with a category named “using mobile” (Table 1) for those taking photos, swiping and playing with mobile phones, as well as calling. Then, the third step was to collect the environmental content of the same activity and the attained spatial elements were obtained to support the necessary spatial content of the activity.

## 3. The Composition and Assessment of the Evaluating Criteria

Compared to normal cities, those residents living in street spaces of high-density cities have completely different spatial experiences and needs, where limited spatial content results in a reduction to those contents of which some are sensitive and others weakened with regard to the residents’ needs. For instance, people are more alerted to avoid cars, which rely more on pillars and railings in the street. Traditionally, people show gathering activity more easily on street corners. Walls along the street are also a very frequent place for gatherings and these gatherings are commonly accompanied with small commercial activities. In addition, slowing the pace, street interfaces and sounds could increase the level of attracting residents that are the common changes that happen ordinarily on streets. Most of these changes are closely related to narrow and short street spaces.

Consequently, it is necessary to implement a specific evaluation system of the healing environment in the street spaces by applying Analytic Hierarchy Process (AHP) statistical models for testing, in order to promote street spaces as ideal health and social spaces for residents. Most of the research data are regarded as multi-hierarchical because its content is composed as the first prior level for constructing the healing environment, and the second level uses research data as the basis. Through different types of activities and a supportive environment, it develops the content of the space for building up the target layer for the street spaces. Based on this basis, it classifies layer-by-layer according to the content of the target layer, defining healing design contents that are appropriate for Macao’s street spaces.

### 3.1. Establishment of the Analysis Index System of Analytic Hierarchy Process

By applying AHP methods, three levels (A, B, C) are used in the evaluation criteria in the system. This is an overall planning principle followed by existing research with references to the relevant literature [40]. Based on the principles of scientific validity, feasibility, hierarchy, relationship, systematic, representativeness, etc., it establishes different evaluation criteria for the system. At the same time, the research methods of similar studies are also referred to [41,42,43]. The evaluation criteria can be referred to in Table 2.

### 3.2. Weighting Determination of the Index System of Analytic Hierarchy Process

In order to quantify the relative importance of each influencing factor, the study employs Saaty’s scaling method in the process of judging a constructing matrix [44]. Digits 1–9 and their reciprocals use are used for the scaling. The professional term “Judgement Matrix” refers to comparing the relative importance of certain factors at higher levels with other related factors (see Table 3). The scale values shown in Table 2 are used to judge and express the relative importance of each factor on each level. U_ij_ is used to represent the importance of comparison between factors i and j, expressed in the form of a judgment matrix.

According to the scale of the Judgment Matrix in Table 2, Judgment Matrix A can be obtained as follows:

(1) $$A=\left[\begin{array}{cccc} A11 & A12 & \cdots & A1n\\ A21 & & & A2n\\ \vdots & & & \vdots \\ An1 & An2 & \cdots & Ann \end{array}\right]$$

where *i*j = 1, 2, 3, …, *n* (); A*i*j = 1, A_j__*i*_ = 1/A_*i*j_.

According to the formula below, it is possible to find the eigenvector of the largest eigenvalue of Judgement Matrix A. W corresponds to the eigenvector of the largest eigenvalue, expressed as

W = (W_1_, W_2_, W_3_, … W_n_)^T^ (2)

The obtained W can be used as a normalized weight vector where

(3) $$\bar{w}i=\sqrt[{n}]{\prod_{i=1}^{n}Aij}\;I=1,\;2,\;3,\cdots ,n;\;\mathrm{Wi}=\frac{\bar{w}i}{\sum_{i=1}^{n}\bar{w}i}\;I=1,\;2,\;3,\cdots ,n$$

In the final consistency check employed, the largest eigenvalue of A is then put in

(4) $$\lambda_{max}=\sum_{i=1}^{n}\frac{\left(Aw\right)i}{nw_{i}}$$

to obtain it. In order to evaluate whether a Judgement Matrix could be consistently satisfactory or not, a consistency index, RI, is introduced (usually to be given by practical experience, Table 4).

When

(5) $$\mathrm{CR}=\frac{CI}{RI}<0.1$$

it assures Matrix A is a consistent matrix; when CR > 0.1, the Judgment Matrix needs to be adjusted until consistency appears. In the formula, CR indicates the ratio of the randomly consistent Judgement Matrix and CI specifies the Judgment Matrix. CI can be obtained from the following formula:

(6) $$\mathrm{CI}=\frac{\lambda_{max^{-n}}}{n-1}$$

### 3.3. Fuzzy Comprehensive Evaluation

In this study we transformed the traditional qualitative analysis into quantitative analysis through the fuzzy comprehensive evaluation method. A theory of membership and a fuzzy transformation is based on fuzzy mathematics [45,46], and this method is suitable for solving various uncertain problems. The following procedures will be set:

1. To determine the evaluation set: The healing environment evaluation of the street space is divided into five levels: “Poor”, “Relatively Poor”, “General”, “Good” and “Very Good”. The evaluation set thus is determined as V = V_1_, V_2_, V_3_, V_4_ and V_5_, where V_1_ = poor, V_2_ = relatively poor, V_3_ = general, V_4_ = good and V_5_ = very good.
2. To establish the factor set of the evaluation indicators: These indicators are set according to Table 2, U = U_1_, U_2_, U_3_, U_4_ and U_5_, where U_1_ = “Positive things to distracting attention”, U_2_ = “Social/spatial support” U_3_ = “Controllability”, U_4_ = “Eliminating environmental stress factors” and U_5_ = “Stimulating positive feeling”.
3. The determination of the value of the index weights: The determination method of the index weights W has been described in the previous chapters. Due to the limited space, the study will not elaborate on it here.
4. To establish an evaluation matrix: According to the evaluation set, it evaluates each item in U = {U_1_, U_2_, U_3_, U_4_, U_5_}. Consequently, to determine the single factor evaluation vector, obtain R_1_, R_2_, R_3_, R_4_ and R_5_, and establish the evaluation matrix R.
5. To select the synthesis operator (the weighted average operator is selected in this paper), synthesize the fuzzy weight vector A and the fuzzy relation matrix R, and get the fuzzy synthesis result vector C of the evaluated object, C = A^O^R = {a_1_, a_2_,…a_p_}(R = {c_1_, c_2_,…c_m_}
  (7) $$C=A^{O}R=\{a_{1},\;a_{2},\ldots ,a_{p}\}\left[\begin{array}{cccc} r11 & r12 & \cdots & r_{1n}\\ r21 & r22 & \cdots & r_{2n}\\ \cdots & \cdots & \cdots & \cdots \\ r_{m1} & r_{m2} & \cdots & r_{mn} \end{array}\right]$$
  C_*j*_ indicates the degree of membership of the evaluated object as a whole for the fuzzy subset of V_*i*_ level.
6. Analyze the fuzzy comprehensive evaluation result vector to normalize C = {c_1_, c_2_, …, c_m_} and C = {c_1_, c_2_, …, c_n_}, where
  (8) $$C_{n}=\frac{c_{n}}{\sum_{i=1}^{n}c_{i}}$$
  according to the comment set V = {v_1_, v_2_, …v_m_}. According to the principle of subordination, the formula W = B*Cp can get the score of each single comprehensive evaluation index.

## 4. Results

### 4.1. Demographic, Social, Cultural and Economic Factors

**Figure 3:**
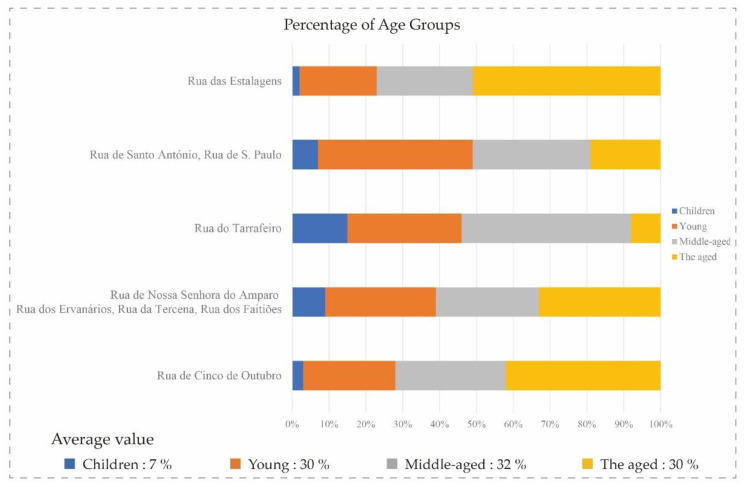
Age distribution of residents in different street spaces.

We graphically analyzed of the use of street spaces and their location, as well as the economic, social and cultural content interacting with this behavior. Figure 3 shows the distribution of various groups of users in terms of age levels. Middle-aged and elderly in the street spaces of Rua das Estalagens street have exceeded 50% of the total number of users, and the number of children is the smallest. In the street spaces of Rua de Santo António and Rua de S. Paulo, most of the users are youths and the next are middle-aged people. In turn, Rua do Tarrafeiro has the largest number of middle-aged users. Besides, the largest number of users of the street spaces in Rua de Nossa Senhora do Amparo, Rua dos Ervanários, Rua da Tercena and Rua dos Faitiões was the elderly group, and the most of users in Rua de Cinco de Outubro was elderly, too. From the statistical data, the elderly users are accounted for as the highest proportion in the study area.

**Figure 4:**
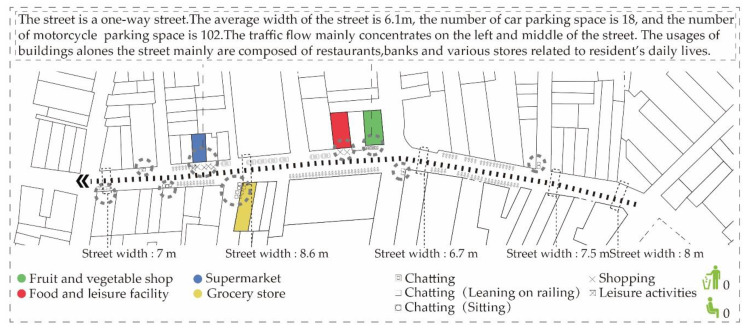
Rua de Cinco de Outubro.

From the survey data in Rua de Cinco de Outubro (Figure 4), it was found that the main participants of the space are middle-aged and elderly groups, and the content of commercial activities mainly includes sales of clothing, daily necessities, large supermarkets, small shops, vegetable shops and restaurants. There are various gathering activities in this space; however, due to the mixed traffic of people and vehicles, and there is no traffic line on the ground, many unsafe factors are added to the lively street scene. People mainly develop gathering activities and behaviors in some corner spaces and concave spaces (the gap between door openings, window sides and motorcycle parking), including sitting and chatting, sitting and taking care of children, standing and chatting, as well as standing around the shop and chatting.

**Figure 5:**
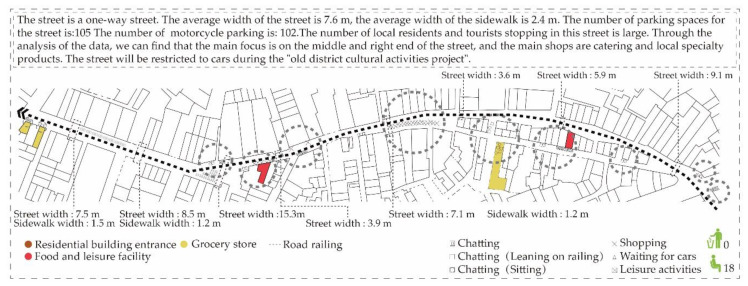
Gathering activities at Rua dos Ervanários, Rua de Nossa Senhora do Amparo, Rua da Tercena, Rua do Minho and Rua dos Faitiões.

The survey data in Rua dos Ervanários, Rua de Nossa Senhora do Amparo, Rua da Tercena, Rua do Minho and Rua dos Faitiões (Figure 5) show that the main participants of the space are the youth, middle-aged and elderly groups. The business activities are mainly bars, daily necessities, small shops, specialty product stores, beverage stores, restaurants, antique shops and stalls. Besides, the street space is also used for various exhibitions at certain times by the government and the streets are closed at the time only for walking. The main participants of these various gathering activities are tourists. Most of the behaviors in these gathering activities are sitting, drinking and chatting, standing and chatting, taking photos, sitting and playing cards, sitting and taking care of children, sitting in the sun and looking at the stalls.

**Figure 6:**
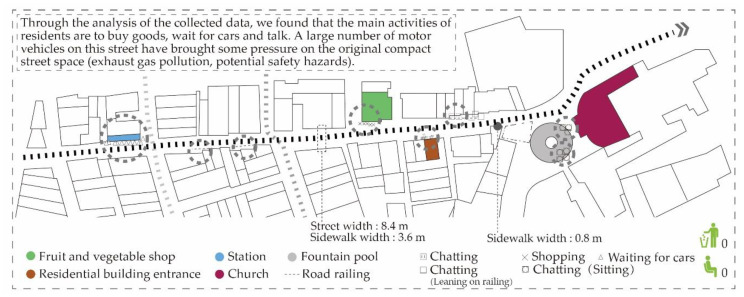
Gathering activities at Rua do Tarrafeiro.

Figure 6 indicates that the main participants of the street space are children, youths and those of middle-age, and most of the commercial activities are fruit shops, daily necessities, beverage shops, restaurants, stalls, etc. Due to the influence of nearby schools and many students around, the various agglomeration activities appear more abundant, including standing and chatting, waiting for the bus, leaning for rest, standing around the fruit shop and chatting, as well as watching the stalls.

**Figure 7:**
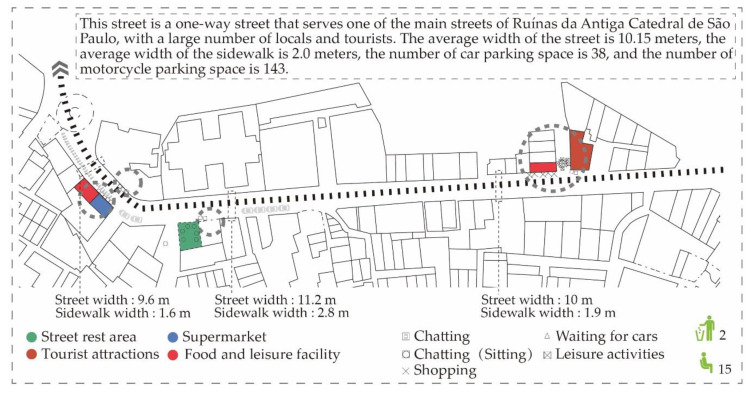
Gathering activities in Rua de Santo António, Rua de S. Paulo.

From the survey to Rua de Santo António and Rua de S. Paulo (Figure 7), it was found that the main participants of the space are children, youths, the middle-aged the and elderly groups, and the commercial activities mainly include supermarkets, specialty product stores, drink shops, restaurants and antique shops. The street space is connected to the main attractions and various agglomeration activities in the space are abundant. Tourists are regarded as the main participants and the main agglomeration activities and behaviors are standing and chatting, taking photos and leaning for rest. In addition, there is a street-side rest area where elderly groups usually carry out activities.

**Figure 8:**
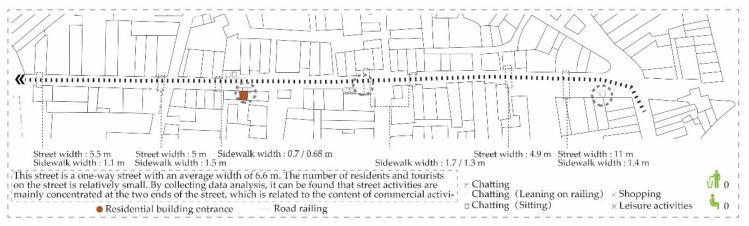
Gathering activities at Rua das Estalagens.

Rua das Estalagens has the highest value among the data obtained from the space syntax. However, according to the field research (Figure 8), the main participants of this space are the youth, middle-aged and elderly groups, and occasionally tourists. Commercial activities on the street include mainly electrical appliances sales, product repairs, etc. Business activities are not prosperous due to some stores being out of business. Besides, most of the street space is occupied by motor vehicles and several sections only provide one-way sidewalks where elderly groups are often the users.

In general, these locations are not significantly different in terms of the personal characteristics of the residents. Macao itself is a city where Western and Chinese cultures are intersecting, which may reflect to the use of street space. The use of street space is not only influenced by physical modes, such as connectivity, aggregation, and accessibility, but also by economic patterns. In the extremely limited street space, residents strive to find space where they can get rest or carry out social activities. The group size is 3–5 people, 5–8 people, scattered, and there are no large gathering groups.

There are also several points that need to be emphasized. The on-site observation found that in the originally narrow streets, most of the street spaces are occupied by cars and motorcycles, resulting in squeezed sidewalk space where some of the pedestrians can have only a distance of 0.5 m with others. The width of the sidewalk in the district should be around 5–7 feet (1.5−2.1 m), but many streets are disconnected and there is almost no room for those in need for the services of public facilities, such as the blind, the disabled, children, pregnant women and the elderly. Besides inadequate public sanitation facilities and street furniture, there are also obvious problems, such as trash bins, resting and sheltering, hand washing tables and benches. As a result, the tight street space, unsafe driving factors, and exhaust gas generated by passing vehicles directly endanger the health of the residents.

### 4.2. Different Spatial Behavior and Its Surrounding Environment Analysis

Referring to the technical details, policy plans and related practical cases in the “Global Street Design Guide” [47], “Urban Street Design Guide” [48], “2040 Seoul Plan” [49], “2030 Seoul Plan” [50] and “Tokyo’s Urban Design” [20], as well as the basis of the field observations and the analysis of the behavioral mapping, these could identify residents’ activities in the space and the conditions needed to develop these activities. Based on the results of the analysis, it helps to carry out reasonable updates and related designs, such as benches, trash cans, rest platforms, parasols, etc., and arranging them according to residents’ activities and health needs [51].

**Figure 9:**
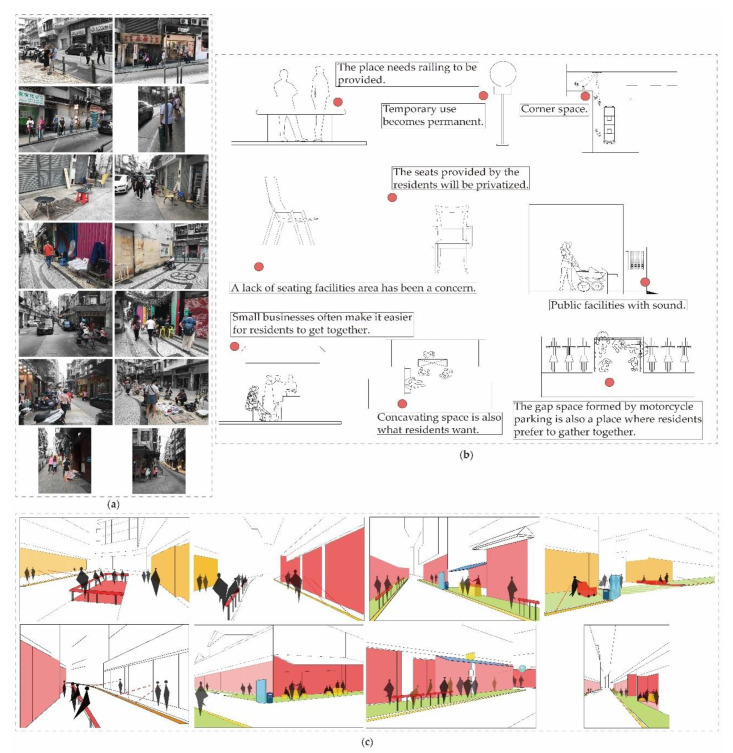
Analytic diagram of different spatial behavior and its surrounding environment. (**a**) Street view; (**b**,**c**) Sketches.

In accordance with these steps, the identification of spatial content and the assessment of accessibility were carried out within the area of research. The characteristics of those spaces that are easy to stay in are semi-enclosed spaces with leaning objects, spaces with commercial activities and spaces with easy visual accessibility. These spaces are all regarded as “informal”, and some of these spaces gradually form permanent spaces from their initial temporary uses. Personal space can be reckoned mostly as resting on streets on railings, warning signs, etc., becoming the main support and leaning objects. Multi-person gathering spaces can be regarded as those streets with rich street types, such as street walls, door openings, street corners, motorcycle parking where the spatial scale is not big, stools, sunshades, etc. becoming the key supporting content (Figure 9).

### 4.3. Data of the Hierarchical Analysis Weighting Index and Fuzzy Comprehensive Evaluation Analysis

#### 4.3.1. Data of the Hierarchical Analysis Weighting Index

Constructing the judgment matrix (see Table 5).

Aiming at positive things to distract attention, namely social/spatial support, controllability, eliminating environmental stress factors and stimulating positive feelings, a total of 5 items were constructed to create a 5-order Judgment Matrix for the AHP hierarchy studies. The eigenvectors were 1.750, 0.580, 0.348, 0.580 and 1.743, and the weighting values corresponding to the range of 5 items were 34.991%, 11.593%, 6.967%, 11.598% and 34.850% (Table 6). In addition, the maximum feature root (5.000) was calculated by combining the feature vectors, and the CI value (0.000) was calculated by using the maximum feature root value (CI = (maximum feature root − *n*)/(*n* − 1)); the CI value is used for the consistency check, as described below.

The CI value is 0.000 for the 5th order of Judgment Matrix and 1.120 for the RI value, so the CR value is 0.000 < 0.1, which means that the Judgment Matrix of this study meets the consistency test and the calculated weight is consistent (Table 7).

#### 4.3.2. Judgment Matrix and Results of the Fuzzy Comprehensive Evaluation

By calculation, the weights of each index of street space healing environment in high-density cities (the old urban areas of Macao) were obtained (Table 8).

(9) $$R11=\left[\begin{array}{ccccc} 0.57 & 0.36 & 0.07 & 0.00 & 0.01\\ 0.01 & 0.20 & 0.29 & 0.33 & 0.18\\ 0.02 & 0.05 & 0.07 & 0.49 & 0.37\\ 0.15 & 0.11 & 0.38 & 0.24 & 0.12\\ 0.06 & 0.20 & 0.32 & 0.26 & 0.16 \end{array}\right]$$

(10) $$R21=\left[\begin{array}{ccccc} 0.16 & 0.18 & 0.34 & 0.21 & 0.11\\ 0.10 & 0.26 & 0.41 & 0.13 & 0.10\\ 0.06 & 0.28 & 0.36 & 0.18 & 0.13\\ 0.12 & 0.33 & 0.31 & 0.17 & 0.08\\ 0.22 & 0.32 & 0.38 & 0.08 & 0.00\\ 0.20 & 0.17 & 0.19 & 0.26 & 0.18\\ 0.24 & 0.49 & 0.23 & 0.04 & 0.00 \end{array}\right]$$

(11) $$R31=\left[\begin{array}{ccccc} 0.12 & 0.14 & 0.39 & 0.23 & 0.13\\ 0.13 & 0.11 & 0.37 & 0.27 & 0.13\\ 0.26 & 0.18 & 0.32 & 0.13 & 0.11\\ 0.17 & 0.29 & 0.36 & 0.09 & 0.09 \end{array}\right]$$

(12) $$R41=\left[\begin{array}{ccccc} 0.06 & 0.42 & 0.34 & 0.12 & 0.06\\ 0.11 & 0.38 & 0.28 & 0.13 & 0.11\\ 0.24 & 0.26 & 0.24 & 0.18 & 0.08 \end{array}\right]$$

(13) $$R51=\left[\begin{array}{ccccc} 0.08 & 0.39 & 0.28 & 0.15 & 0.11\\ 0.35 & 0.27 & 0.21 & 0.13 & 0.04\\ 0.04 & 0.41 & 0.36 & 0.11 & 0.08 \end{array}\right]$$

We use AHP to obtain the weight distribution vector of each index factor, i.e., W = (0.35, 0.12, 0.07, 0.12 and 0.35). Through conducting on-site questionnaire surveys and collecting the data, the evaluation vector of each individual factor was finally obtained, which was calculated by using the M(.,+) operator:

R1 = U1i * R1 = (0.06, 0.06, 0.39, 0.12, 0.38) * R1 = (0.085, 0.142, 0.213, 0.336, 0.225)(14)

R2 = U2i * R2 = (0.11, 0.23, 0.09, 0.07, 0.09, 0.30, 0.12) * R2 = (0.161, 0.263, 0.301, 0.169, 0.105)(15)

R3 = U3i * R3 = (0.16, 0.25, 0.49, 0.10) * R3 = (0.196, 0.167, 0.346, 0.176, 0.116)(16)

R4 = U4i * R4 = (0.47, 0.47, 0.05) * R4 = (0.093, 0.393, 0.306, 0.125, 0.084)(17)

R5 = U5i * R5 = (0.49, 0.30, 0.21) * R5 = (0.150, 0.359, 0.275, 0.133, 0.082)(18)

The evaluation matrix R is obtained from R1, R2, R3, R4 and R5:

(19) $$R=\left[\begin{array}{ccccc} 0.085 & 0.142 & 0.213 & 0.336 & 0.225\\ 0.161 & 0.263 & 0.301 & 0.169 & 0.105\\ 0.196 & 0.167 & 0.346 & 0.176 & 0.116\\ 0.093 & 0.393 & 0.306 & 0.125 & 0.084\\ 0.150 & 0.359 & 0.275 & 0.133 & 0.082 \end{array}\right]$$

U = (U1, U2, U3, U4, U5) = (0.35, 0.12, 0.07, 0.12, 0.35)(20)

(21) $$R^{\left(A\right)}=U*R=\left(0.35,\;0.12,0.07,0.12,0.35\right)*\;\left[\begin{array}{ccccc} 0.085 & 0.142 & 0.213 & 0.336 & 0.225\\ 0.161 & 0.263 & 0.301 & 0.169 & 0.105\\ 0.196 & 0.167 & 0.346 & 0.176 & 0.116\\ 0.093 & 0.393 & 0.306 & 0.125 & 0.084\\ 0.150 & 0.359 & 0.275 & 0.133 & 0.082 \end{array}\right]$$

After normalizing the calculation results, R(A) = (0.125, 0.263, 0.265, 0.210, 0.137) was obtained. First, the evaluation index weight vector matrix A and the 5 * 5 weight judgment matrix R were constructed, and finally the weight values of the five comment sets were analyzed: 0.125, 0.263, 0.265, 0.210 and 0.137. From the above data, it can be reckoned that the weight value of V3 in the five comment sets is the highest (0.265). According to the rules of maximum membership, the final comprehensive evaluation result is “general”. The results showed that the healing environment of the street space was rated as “general” 0.265, “relatively poor” 0.263, “good” 0.210, “very good” 0.137 and “poor” 0.125. According to the principle of maximum membership, the healing environment of the street space in this area is at a “general” and “relatively poor” level, which is relatively consistent with the actual situation.

## 5. Discussion and Conclusions

### 5.1. Discussion

According to the principle of maximum membership, it can be estimated that the healing environment of the street space in Macao is at a “general” level. Membership in the “relatively poor” level is 0.263, and the two values are remarkably close. Although the degree of subordination to “very good” is only 0.137, from a positive point of view, this indicates that there is still a lot of room for improving the capacity of the healing environment. Based on the quantified data, it reveals the current actual evaluation of residents in the space environment, and the most important aspect is that the results can clarify the primary and secondary relationships of the improvement points and provide supportive data for the arguments proposed. In general, by analyzing the weight of the indicators from the standard level, it can be clearly seen where residents think that distracting positive things and inspiring positive feelings have a greater impact (the weight is 0.35). However, social/spatial support and eliminating pressure on the environment was weighted as 0.12, and controllability weighted as 0.07, which did not provide good fuzzy evaluation results, and they were both at the “poor” level and close to the “relatively poor” level. This indicates that residents cannot obtain a good healing perception when using existing street spaces.

The main purpose of this research is to investigate the use of residents in the street space of high-density cities in Macao and the spatial content that supports various types of usage activities, thereby constructing the content of the healing environment of the street spaces. The study used multi-level models and fuzzy evaluation to analyze the status of street space usage from resident survey data, which revealed a more intuitive understanding of the current status of the healing environment of the street spaces based on the values generated by the model, and put forward corresponding countermeasures to the existing problems. The goal is to improve the environment of the street spaces in Macao, which would be beneficial to the local city managers and designers, through enriching the research content of what constitutes a healing environment. The realization of the healing environment in street spaces is particularly important for Macao. It could enhance the vitality of the space, so that residents, especially the elderly residents, can get a higher level of physical and mental health, as well as improving their sense of belonging; this way, the overall public health level of Macao can be built for social equity, promoting social sustainability and an environmentally friendly economy.

Behavioral data analysis showed that there are more than 50% of the total users of the elderly in the street spaces of Rua das Estalagens and 40% of the elderly in Rua de Cinco de Outubro. By referencing spatial accessibility and the survey of business environment, the commercial activities of this street are mostly catering as well as fruit and vegetable markets, which further reflects with the content of the residents’ daily interactions, closely related to the number of middle-aged and elderly in the spaces. According to the recording and analysis of the space supporting the environment, the spatial content that is conducive to the participation and use of residents in the space is then defined. Therefore, there are many focus points for projects to resolve problems, such as the void generated by irregular parking of motorcycles, corner shops, street rest space, railings, columns and those happening often in street spaces. In the hierarchical analysis and fuzzy evaluation of the healing environment, the overall evaluation is “general”, indicating the current situation where the analysis and suggestions are made accordingly.

There are several following points that should be paid attention to during the construction of a street space as a healing environment in Macao:

1. Social/spatial support: Indirectly, it indicates that managers pay insufficient attention to the configuration and renewal of various facilities in spaces, and the lack and uneven distribution of public health facilities result in a poor experience for the overall public health. Due to the limited space, most of the facades along the street are occupied by commercial billboards, and some of them are dilapidated, causing a certain impact to the spatial visual experience of residents and tourists. The shortage of public facilities for children, the elderly and the disabled may reduce the capability of group activities in street spaces and cause an unfairness to space use. The corner space is also reckoned as an important space for residents to gather, but their presence usually found in this research is closely related to the business environment. However, there remains certain exclusivity in some corner spaces and residents’ demand for corner spaces has been confirmed that it is more likely to produce a sense of security and a good observation of the surrounding environment, revealing the reasonable planning, construction and maintenance from related departments of government.
2. Eliminating the stress factors in the environment as one of the factors of building the healing environment: if its physical comfort and psychological comfort evaluation indices are indicated as “poor”, it is highly connected with the high-density spatial heterogeneity of the space. For example, the lack of plants and water could reduce residents’ perception of health and show a lack of novelty of the street spaces for existing residents. Consequently, spatial renewal and design work has been very urgent. Apparently, residents are tired of problems caused by inappropriate planning, such as the occupation of motorcycle parking in the street and most of these motorcycle’s users are the young people whose dependency on the street spaces is relatively weak compared to the elderly.
3. The controllability of the street space is described as “poor”, which is caused by the mixture of pedestrian and traffic vehicles between the streets; such situations are unsafe to the residents. It needs effective communication on planning and management by the traffic authorities, especially for the most efficient use of the street space by getting rid of illegal users. When spatial lighting and visual accessibility are at the general level, the visual perception could not be better improved due to the influence of the spatial forms of the different streets. It needs to strengthen the improvement of street spatial infrastructure by governmental managers to ensure the safety of the residents at night.
4. Distraction via positive things, for urban residents and other users of the space: all kinds of positive events and interesting things in the street space is an effective way to improve resident’s participation and usage with an enthusiastic attitude. Social interaction and shopping behavior are reflections of spatial viability. Therefore, it is needed to consider the promotion of activating activities for increasing spatial viability while implementing spatial renewal. When the plants in the space are indicated as the “poor” level, and due to limited space as they need a large area and not hard pavement, the improvement of applying vertical greening on building facades and roof gardening can be an effective solution to encourage and support residents with enthusiasm to participate in planting in order to relieve the dependence on government management. However, when carrying out vertical greening of a street space, attention should be paid to the damage caused by heavy rainfall every year. Government must conduct suitable planning for implementing vertical greening that can cultivate residents’ awareness of participation in planting. More importantly, it helps residents realize that effective planting can protect their own health and the rights of using the street spaces, as well as to ensure the provision of greenery for better street environments.
5. Stimulating positive feelings in the fuzzy evaluation was indicated as “general”, but from the aspect of the micro environment of climate, the perception of physical and mental health remains in the “relatively poor” category, and the opportunity to relax indicated as “poor”. For those factors shown previously, it is reckoned that when most of the problems are resolved or improved, users of the street space could receive more positive feelings.

In view of the above problems, suggestions for solving related projects are put forward:

1. Distracting positive things:
  - Adjust the street space through a phased method, using short-term materials for the short-term, after the public understands and passes the test, randomly using permanent materials to replace them.
  - The street is a functional space, it is also an economic asset, and economic behavior events such as shopping in the space will bring higher profits and vitality.
  - Traffic accidents are one of the biggest hidden safety hazards in the space, and the transportation department will jointly assign people and vehicles to the streets with high pedestrian flow, separately, and provide safety warning pavement devices.
2. Social/space support:
  - To prevent private occupancy of the space.
  - Installing street furniture, including separation pillars and benches.
  - The sidewalk width should be 1.5 m (but current street spaces are not possible to fulfill such width).
  - Adding spatial lighting and shading facilities to ensure safe and sheltered spaces for users.
  - Fixing problems of existing accessibility.
  - Setting pedestrian ramps at pedestrian crossings where the level changed and applying non-slip material on them.
  - Public sanitary facilities, such as fountain-type water dispensers, hand-washing tables and trash bins, assure the use of those spaces with hygiene and pleasure.
3. Controllability:
  - Providing sufficient lighting for dark and narrow street spaces that are prone to crime and using transparent metal shutters instead of the closed ones for those stores with hidden safety issues.
  - Adding rest areas in streets by replacing public rest platforms with parking spaces and the same width of the ideal street rest area and parking lanes, considering accessibility and visibility by applying open guardrails for defining spaces.
4. Eliminating stress factors in the environment:
  - Street spaces occupied by cars and motorcycles can be remodeled segmentally as the curb extension belt (“narrow point type”) at the middle section of the road. It is possible to implement the plan of shared motorcycles to reduce the occupancy of private motorcycles by setting public sharing spaces.
5. Inspire positive feelings:
  - Landscaping to be employed to improve the microclimate and reduce the urban heat island effect felt in the street spaces.

### 5.2. Conclusions

This study provides a new understanding of the street renovation needed for high-density cities in Macao, based on the concept of a healing environment. The results could help city managers and designers to design street spaces that are more conducive to residents’ use, being safe and healthy spaces. To assess the impact of policy decisions related to the street space environment on health, it is necessary to understand the complex relationship between various related factors through multiple channels. The “bottom-up” research method and systematic research perspective provide policy makers with reference predictions of expected and unexpected results. In reality, residents’ street behavior is affected by the space environment. If these effects are positive, then residents can obtain physical and psychological health content when using it, showing social health with positive significance. The results of the study point out the current status and problems of the use of street space in the old urban areas of Macao, and we propose relevant solutions to specific problems. Therefore, designers should consider these strategies when designing updates; however, this study has some limitations. First, due to the limited time and human resources, these observations cover only one season. Since the content of the activities on the streets may be altered differently in different seasons, it is recommended to conduct further research in different seasons. Secondly, the study area is an old city, which has its spatial characteristics, but also has certain limitations. It is suggested that the study can be carried out again in other urban street spaces, showing its appropriateness for constructing a healing environment for cities. As a result, future research should explore the other influencing factors that influence the construction of such healing environments in streets, to strengthen the theoretical and scientific basis of street space health design practices for Macao’s urban renewal. It also provides references for the study of street spaces in tropical and subtropical cities with similar urban cultural backgrounds, such as other high-density cities in Asia.

## Figures and Tables

**Table 1:** Statistics of each behavior category.

Behavior Category	Content	Frequency
Standing	Watch	55
	Taking pictures	307
	Conversation	217
	Waiting for the bus	312
	Shopping	95
Sitting	Rest	261
	Conversation	28
	Sale of goods	16
Exercise	Running	5
Relax	Look around	11
	Eat	15
	Drink	21
Rely on	Waiting for bus	32
	Rest	68

**Table 2:** The environmental hierarchy structure system of the healing environment in the street spaces.

Objective Level	The First-Level Indicator Level (U)	The Second-Level Indicator Level
Healing environment in street spaces of a high-density city	Positive things to distracting attention (U_1_)	Plants
		Social interaction
		Shopping behavior
		Spatial interest
		Pedestrians or events in the streets
	Social/spatial support (U_2_)	Street railing
		Building walls
		Street corner
		Public health facilities
		Shade and rain shelter
		Pillar (electric poles, warning signage)
		Children, aged, disabled facilities
	Controllability (U_3_)	Lighting
		Visual accessibility
		Street spatial navigation system
		Spatial selections (safe, quiet or noisy area)
	Eliminating environmental stress factors (U_4_)	Physical comfort
		Psychological comfort
		Spatial occupancy of car and motorcycle
	Stimulating positive feeling (U_5_)	Microclimate environment
		Opportunity of rest and relax
		Perception of physical and mental health

**Table 3:** The Judgement Matrix.

Scale	Degree of Importance of U_*i*_ in Comparison to U_j_
1	The two factors are equally important
3	One factor is slightly more important than the other is
5	One factor is significantly more important than the other is
7	One factor is substantially more important than the other is
9	One factor is fundamentally more important than the other is

Note: 2, 4, 6 and 8 denote the intermediate values of the adjacent judgments of U_*i*_ and U_j_.

**Table 4:** Average random consistency RI index.

*n*	1	2	3	4	5	6	7	8	9
**RI**	0	0	0.58	0.90	1.12	1.24	1.32	1.41	1.45

**Table 5:** Criterion comparison matrix.

Item	Positive Things to Distracting Attention	Social/Spatial Support	Controllability	Eliminating Environmental Stress Factors	Stimulating Positive Feeling
Positive things to distracting attention	1	3	5	3	1
Social/spatial support	0.33	1	1.67	1	0.33
Controllability	0.2	0.6	1	0.6	0.2
Eliminating environmental stress factors	0.33	1	1.67	1	0.33
Stimulating positive feeling	1	3	5	3	1

**Table 6:** Criterion level judgment comparison result.

Item	The Feature Vectors	Weight Value	Maximum Eigenvalue	CI Value
Positive things to distracting attention	1.750	34.991%	5.000	0.000
Social/Spatial support	0.580	11.593%		
Controllability	0.348	6.967%		
Eliminating environmental stress factors	0.580	11.598%		
Stimulating positive feeling	1.743	34.850%		

**Table 7:** Summary of the consistency test results at the criterion level.

Summary of Conformance Test Results				
The Largest Eigenvalue	CI Value	RI Value	CR Value	Consistency Test Results
5.000	0.000	1.120	0.000	Pass

**Table 8:** Summary table of the weights and comments.

The First-Level Indicator	Weight	The Second-Level Indicator	Weight	V_1_	V_2_	V_3_	V_4_	V_5_
Positive things to distracting attention U_1_	0.35	Plants U_11_	0.06	0.57	0.36	0.07	0.00	0.01
		Social interaction U_12_	0.06	0.01	0.20	0.29	0.33	0.18
		Shopping behavior U_13_	0.39	0.02	0.05	0.07	0.49	0.37
		Spatial interest U_14_	0.12	0.15	0.11	0.38	0.24	0.12
		Pedestrians or events in the street U_15_	0.38	0.06	0.20	0.32	0.26	0.16
Social/ Spatial support U_2_	0.12	Street railing U_21_	0.11	0.16	0.18	0.34	0.21	0.11
		Building wall U_22_	0.23	0.10	0.26	0.41	0.13	0.10
		Street corner U_23_	0.09	0.06	0.28	0.36	0.18	0.13
		Public health facilities U_24_	0.07	0.12	0.33	0.31	0.17	0.08
		Shade and rain shelter U_25_	0.09	0.22	0.32	0.38	0.08	0.00
		Pillar (electric pole, warning sign) U_26_	0.30	0.20	0.17	0.19	0.26	0.18
		Children, aged, disabled Facilities U_27_	0.12	0.24	0.49	0.23	0.04	0.00
Controllability U_3_	0.07	Lighting U_31_	0.16	0.12	0.14	0.39	0.23	0.13
		Visual accessibility U_32_	0.25	0.13	0.11	0.37	0.27	0.13
		Street space navigation system _U33_	0.49	0.26	0.18	0.32	0.13	0.11
		Spatial selections (safe, quiet or noisy area) U_34_	0.10	0.17	0.29	0.36	0.09	0.09
Eliminating environmental stress factors U_4_	0.12	Physical comfort U_41_	0.47	0.06	0.42	0.34	0.12	0.06
		Psychological comfort U_42_	0.47	0.11	0.38	0.28	0.13	0.11
		Spatial occupancy of car and motorcycle U_43_	0.05	0.24	0.26	0.24	0.18	0.08
Stimulating positive feeling U_5_	0.35	Microclimate environment U_51_	0.49	0.08	0.39	0.28	0.15	0.11
		Opportunity of rest and relax U_52_	0.30	0.35	0.27	0.21	0.13	0.04
		Perception of physical and mental health U_53_	0.21	0.04	0.41	0.36	0.11	0.08
